# NEWS

**DOI:** 10.7189/jogh.05010202

**Published:** 2015-06

## BILL AND MELINDA GATES FOUNDATION

➢ Bill and Melinda Gates’ annual letter, published in January 2015, outlined their hopes for the next 15 years. It contained some far–reaching predictions on public health, where they predict by 2030 four more diseases will join smallpox as being completely eradicated. The most likely candidates are polio, Guinea worm, elephantiasis, river blindness and blinding trachoma. “The drugs that can stop these scourges are now being donated in huge numbers by pharmaceutical companies, and they’re being used more strategically thanks to advances in digital maps that show where diseases are most prevalent,” the letter states. (*Business Insider*, 22 Jan 2015)

➢ Bill Gates praised aid efforts to countries affected by Ebola, but warned that the response would have been inadequate had Ebola been more infectious. Now that the epidemic appears to be abating, he calls for countries and aid agencies to “respond faster” by ensuring that volunteer lists are available quickly, and using experimental drugs at an earlier stage to counteract other, potentially more infectious, pathogens. He also announced the new “Global Citizen” social network for volunteers and activists to become more involved in the fight against global poverty. (*Financial Times*, 22 Jan 2015)

➢ The BMGF pledged to invest US$ 52 million in the German biopharmaceutical company CureVac. CureVac will use this investment to develop its proprietary mRNA platform, and to collaborate on the development of vaccines for diseases which disproportionately affect poorer countries. CureVac is investigating the use of mRNA as a data carrier to instruct the human body to produce its own proteins to fight diseases. The technology can potentially allow for the rapid and cost–effective production of multiple drugs and vaccines which are thermo–stable – eliminating the need for cold storage and making them more suitable for developing countries. The agreement’s terms mean that CureVac must set an affordable price for any BMGF–funded products in developing countries. (*European Biotechnology News*, 10 Mar 2015)

➢ In an editorial published in the *New England Journal of Medicine*, Bill Gates warns that the world must learn from the lessons of Ebola, as the crisis fades from attention. Inadequate basic preparation caused delays in combating Ebola, which could be catastrophic if there is an outbreak of a more dangerous disease. He calls for a global warning and response system for outbreaks, coordinated by a sufficiently empowered and funded global institution. Such a system should: enable speedy global decision making; increase R&D investment and clarify regulatory pathways; improve early warning and detection systems; involve trained staff and volunteers; strengthen health systems in low– and middle–income countries; and incorporate preparedness exercises to identify shortcomings. (*New England Journal of Medicine*, 18 Mar 2015)

➢ Bloomberg Philanthropies and the BMGF have launched a global fund to help developing countries fight legal challenges to their smoking laws by the tobacco industry. It was launched to counteract the industry’s apparent moves to block anti–smoking legislation in developing countries – who often lack resources to defend legal challenges. Health experts claim this has gathered speed as smoking rates fall in developed countries, and the industry seeks to maintain its hold in developing countries – home to 75% of the world’s smokers. The fund, administered by the Campaign for Tobacco–Free Kids, will also help countries to draft tobacco legislation that will avoid legal challenges. Tobacco companies say that there are few active cases, and it is routine to give a legal opinion when interests are affected by a proposed law. (*New York Times*, 18 Mar 2015)

## GAVI ALLIANCE

➢ In a bid to spur development of an Ebola vaccine, GAVI has committed US$ 300 million to buy the vaccine (conditional upon WHO recommendation), and may spend another US$ 90 million to offer immunisation. The Ebola outbreak has led to debates on the lack of treatment options, and the need to encourage the pharmaceutical industry – wary of investing in Ebola treatments due to limited commercial opportunities – to commit more to develop therapies for tropical diseases. This has been consistently highlighted by the WHO, and in another response, the US government will offer immunity against any legal claims relating to the development and roll–out of three Ebola vaccinations under development. (*Wall Street Journal*, 12 Dec 2014)

➢ GAVI’s fund–raising campaign to buy 2.7 billion vaccine doses against diseases such as diphtheria, diarrhoea and polio is being undermined by the strength of the US dollar and the surging Swiss franc. It had set a target of raising US$ 7.5 billion to purchase these doses, but these unforeseen exchange rate movements, which occurred after its initial requests, mean that it may not be able to buy the full amount of doses. The dollar has strengthened against most currencies since summer 2014, making eg, pledges made in euros less valuable. For example, GAVI buys many vaccines from pharmaceutical firms in Switzerland, and the surging Swiss franc (which occurred after the Swiss central bank stopped pegging it to the euro) has serious implications for GAVI’s plans. GAVI will negotiate with the pharmaceutical companies if it faces a shortfall. (*The Guardian*, 23 Jan 2015)

➢ The US pharmaceutical company Pfizer announced that it will cut the price of its pneumococcal vaccine Prevenar 13 by 6% to US$ 3.10/dose in poor countries, as part of its commitment to the GAVI global vaccines alliance. This reduction will last until 2025, safeguarding access against economic growth in developing countries. This announcement came ahead of GAVI’s fundraising conference in Berlin, where it hoped to raise US$ 7.5 billion to extend immunisation in the developing world between 2015 and 2020. GlaxoSmithKline also extended its price–freeze commitment by 10 years for countries moving on from GAVI support, and Sanofi agreed to increase production of yellow fever vaccines to address severe shortages. (*Yahoo news*, 26 Jan 2015)

➢ Bangladesh will introduce two new vaccines into its national immunization programme, with the support of GAVI, UNICEF, WHO and the Global Polio Eradication Initiative. More than 3 million children will receive the pneumococcal vaccine, which protects against the one of the main causes of pneumonia, and the Inactivated Polio Vaccine (IPV). Pneumonia is a major killer in Bangladesh, causing 22% of deaths in children aged under 5 years, and introduction of the vaccine will have a major impact on child mortality. Introducing IPV to routine immunisation will further improve immunity and reduce risks associated with vaccine–derived polioviruses. (*GAVI*, 20 Mar 2015)

➢ For the first time, deaths caused by cervical cancer are set to outstrip deaths from childbirth. This is partly because the number of women dying in childbirth has fallen by nearly 50% since 1990, to 289 000 per year. At the same times, deaths from cervical cancer have risen to 266 000 per year – a 40% increase. Anuradha Gupta, the Deputy CEO of GAVI, states that these deaths are almost entirely preventable by HPV vaccines, screening and treatment. However, almost 90% of deaths from cervical cancer occur in developing countries with scarce screening and treatment services. GAVI has worked with manufacturers to reduce HPV vaccines prices to US$ 4.50 per dose, which will extend coverage across 27 countries. Cancer prevention is much more efficient than treatment, and cervical cancer strikes younger, economically–active women – devastating and impoverishing families. (*Project Syndicate*, 14 Apr 2015)

## WORLD BANK

➢ A global coalition, spearheaded by the World Bank, Rockefeller Foundation and WHO, is campaigning for greater progress towards universal health coverage (UHC). Launching on 12 Dec 2014, it highlighted UHC’s role in saving lives, ending extreme poverty, building resilience against climate change and ending deadly epidemics. This marks the second anniversary of the UN’s resolution which endorsed UHC as a pillar of sustainable development and global security. Each year, 100 million people fall into poverty because of health care costs, and 1 billion people cannot access health care – so disease outbreaks can become epidemics. More low– and middle–income countries are moving towards UHC, recognising that it can protect and improve lives. Knowledge and technologies exist to save and improve lives, but their impact is limited when health care access is limited. (*UHC*, 12 Dec 2014)

➢ The World Bank report *The Economic Impact of Ebola on Sub–Saharan Africa: Updated Estimates for 2015* quantifies Ebola’s economic impact. It found that Ebola caused economic growth for 2014 in Guinea to fall from 4.5% to 0.5%, from 5.9% to 2.2% in Liberia and from 11.3% to 4% in Sierra Leone. Cumulatively, this represents US$ 0.5 billion – nearly 5% of these countries’ GDP. The Bank slashed its 2015 growth forecasts for these countries, to –0.2% for Guinea, 3% for Liberia and –2.0% for Sierra Leone – representing a GDP loss of US$ 1 640 million – more than 12% of their combined GDP. The region is now experiencing wide–spread food insecurity and unemployment. However, the economic impact elsewhere in Africa was limited by the swift response in averting potential outbreaks. The report calls for the speedy elimination of Ebola, and for improved readiness to avoid the human and economic costs of pandemics. (*World Bank*, 20 Jan 2015)

➢ The World Bank is working with the UN, IMF and regional development banks on a new pandemic emergency facility to incentivise poor countries’ readiness for epidemics, and allow for prompt support to countries in crisis. It draws upon three financial elements. First, the existing International Bank for Reconstruction and Development, which provides loans, guarantees and risk management products to low– and middle–income countries. Second, the public contingent funding facility, which provides funding to more developed countries, and facilitates speedy crisis management. Third, a new market–based insurance mechanism is under development. Countries would fulfill their obligations by ensuring stronger health systems and being more prepared for an outbreak, whilst insurers have an incentive to work towards disease prevention. The mechanism includes a plan for tackling future major disease outbreaks, expanding laboratory and drug coverage, and increasing the numbers of health workers, and may be available later in 2015. (*Public Finance International*, 10 Mar 2015)

➢ According to the World Bank economist Christoph Lakner, the ten richest African people have more wealth (worth US$ 62 billion) than the poorest 50% of Africa’s population (worth US$ 59 billion). Similar calculations for India shows that the poorest 50% of its population own the same wealth as the wealthiest 22 individuals; and in China the richest 5 individuals own the same wealth as the poorest 10% of the population – this would cover more than 40% of the poorest African people. (*World Bank Blog*, 11 Mar 2015)

➢ The UK’s Overseas Development Institute (ODI) report *The Data Revolution: Finding the Missing Millions* warns that millions more people could be living in extreme poverty than previously estimated. Due to inadequate data collection, the World Bank estimate of 1.01 billion people living in extreme poverty – defined as living on US$ 1.25/day – could be 25% higher. This means that the number of estimated maternal deaths could be inaccurate, and it is unknown how many people are living in cities, how many girls are married before the age of 18 years, etc. It is impossible to tackle poverty and meet development goals without accurate data, but the authors believe that opening up the World Bank’s database on income distribution, developing new technologies to allow data to be more readily available, and more investment in data collection and analysis would improve data sources. These methods are needed to help the world’s poorest, they conclude. (*The Guardian*, 1 Apr 2015)

## UNAIDS/GLOBAL FUND

➢ 34 nations have adopted the target of eliminating malaria by 2030, with an estimated cost of US$ 8.5 billion. This estimate may be revised if disease control measures change due to response to developments, such as increasing drug and insecticide resistance. This estimate comes ahead of the launch of the WHO’s global strategy for tackling malaria, and ongoing discussions on including malaria as a target in the post 2015 Sustainable Development Goals. According to the WHO, eight countries have eliminated malaria since 2000. However, global aid for malaria has levelled off, and there has been a 20% reduction in funding for these 34 countries from the Global Fund as it focuses more on higher–burden, lower–income countries. (*scidev.net*, 15 Jan 2015)

➢ Nigeria has passed the HIV and AIDS Anti–Discrimination Act 2014, in a move to tackle stigmatisation and discrimination against people living with HIV. As well as preventing discrimination, the legislation enables access to health care and other services, and the protection of the human rights and dignity of HIV–positive people. The UNAIDS Country Director for Nigeria, Dr Bilali Camara, welcomed the legislation, and stated UNAIDS’s commitment to working with the government to support its implementation. It is part of Nigeria’s wider efforts to end the HIV epidemic by 2030, which has seen a 35% reduction in the number of new HIV infections by 2013. Measures include testing 4 million pregnant women and 8.2 million adults in the past four years, and expanding the number of sites providing services to prevent mother–to–child transmission from 675 in 2010 to 5 622 in 2013. (*NACA*, 2 Feb 2015)

➢ The Global Fund has awarded the pharmaceutical company Cipla a US$ 189 million tender for ARV drugs to treat HIV/AIDS. This would enable the drugs – which will be manufactured in India – to be supplied to over 140 countries. Cipla has been associated with the Global Fund since 2002, when it was awarded a long–term contract for supplying anti–malarial drugs. The Global Fund has supported 7.3 million people accessing ARV therapy for HIV, has tested and treated 12.3 million people for TB, and has distributed 450 million mosquito nets to protect against malaria. (*Economic Times India*, 13 Feb 2015)

➢ UNAIDS welcomed Belarus’s confirmation that it imposes no restrictions on the entry, stay and residence for HIV–positive people, and that foreign nationals will have equal access to health services, including HIV treatment. This announcement brings Belarus’s HIV–related laws and policies into alignment with international public health and human rights standards. This moves leaves only three countries in eastern Europe and central Asia with HIV–related travel restrictions. There is no evidence that such restrictions protect public health or prevent HIV transmission. They have no economic justification as HIV–positive people can lead long and productive working lives. “In Belarus and elsewhere, freedom of movement is a right for everyone to enjoy, regardless of HIV status,” says UNAIDS Executive Director Michel Sidibé. He also urged for the lifting of such restrictions in the remaining 37 countries, territories and areas where they still apply. (*UNAIDS*, 9 Apr 2015)

➢ The “low bono” financial model is being deployed the Global Fund to leverage more support from the private sector. Under a “low bono” arrangement, a company charges a below–market rate for a product or service – which can be sufficient to generate sustainability, and is not regarded as a charitable donation. The Global Fund has also created an innovation hub to bring together private sector partners to tackle challenges relating to supply chains, financial and risk management, and programme quality. New financial models are critical for funding the Sustainable Development Goals, and banks could also play a crucial role in capacity building and product development eg, in microfinance. (*Devex*, 15 Apr 2015)

## UNITED NATIONS

➢ UNHCR, the UN refugee agency, reports that fighting in eastern Ukraine’s Donetsk region is creating new displacement, and nearly 1 million people are internal refugees – set to increase as more people are registered. In addition, 600 000 Ukrainians have been displaced to neighbouring countries, eg, Moldova, Poland and Romania, since Feb 2014. Fighting in the Donetsk region has led to the destruction of buildings, infrastructure and basic services. Many recently–displaced people arrive with few belongings and without proper winter clothes. The UNHCR is working with partners to distribute relief such as clothing and bedding. The UNHCR states that the lack of access to public services is exacerbating an already desperate situation, and calls on all parties to refrain from actions that might endanger civilians, and to adhere to international humanitarian law. (*UNHCR*, 6 Feb 2015)

➢ It is difficult to define and measure sustainable development, so UN negotiators have a difficult task in specifying indicators for the Sustainable Development Goals (SDGs) before they are finalised in Sept 2015. There are four key challenges in ensuring that the goals are measurable in a way that sets out a globally sustainable future. First, there are 17 draft goals and 100 indicators but it is unclear who will provide data to monitor and manage their implementation. Second, international bodies and countries need a scientific method for including innovative data sources (eg, mapping technologies) into SDG monitoring and evaluation. Third, there are too many data categories and indicators, eg, transport access indicators do not capture economic, pollution and climate costs. Lastly, there are frequent gaps between what science measures and how policy is designed, eg, air quality indices may communicate risk but not identify steps to improve air quality. (*SciDev.net*, 11 Feb 2015)

➢ At the UN’s Commission on the Status of Women, delegates adopted a declaration that confirmed their commitment to achieving gender equality by 2030. However, women’s rights and feminist groups argue that the declaration did not go far enough towards the transformative agenda needed to achieve gender equality. The executive director of UN Women, Phumzile Mlambo–Ngcuka, stated that progress has been slow, with “serious stagnation and even regression in some areas.” According to UN Women, it will take more than 80 years to achieve economic gender parity, and 50 years to achieve parliamentary gender parity at the current rate of progress. Indigenous women, women with disabilities and women marginalised due to their sexual orientation must be better served by their governments said Mlambo–Ngcuka, adding that substantial steps towards gender equality must be evident within the next 5 years. (*The Guardian*, 10 Mar 2015)

➢ US$ 4 billion has been pledged in humanitarian aid for Syrians, but the UN says this is less than half the amount needed to help those affected by the war. The UN asked for US$ 8.4 billion (US$ 2.9 billion for those inside Syria, and US$ 5.5 billion for those who have fled to other countries). The UN secretary–general, Ban Ki–moon, described the war in Syria as “the worst humanitarian crisis of our time.” Oxfam, the international aid agency, has criticised the “inadequate” international response to the crisis. In addition, the UN’s Financial Tracking Service said in November 2015 that nearly 25% of the previous year’s pledges (US$ 585 million) are unfulfilled. (*The Guardian*, 31 March 2015)

➢ In 2014, 3 300 migrants died attempting to enter Europe, with most drowning in the Mediterranean Sea. 2015 is likely to be worse – according to the UNHCR 60 000 people have crossed the Mediterranean Sea thus far, and 1800 have died. Migrants are fleeing poverty, inequality, climate change, conflicts and human rights violations in Africa and the Middle East. Federica Mogherini, the High Representative of the European Union for Foreign Affairs, called on the international community Security Council to end the crisis and dismantle the people–smuggling networks that support it. Mogherini states that these crossings are security as well as humanitarian crises, as smuggling networks are linked to finance and terrorist activities. The EU is calling for a UN resolution to disrupt networks and destroy boats before their use. The UN Secretary–General Ban Ki–moon rejected a military operation as “potentially dangerous for migrants and local fishermen.” (*IPS*, 11 May 2015)

## UNICEF

➢ *Sure Chill*, a small UK company located in a remote area of Wales, has designed a fridge that can run for 35+ days without electricity. Its main aim is to protect vaccines from damage caused by patchy refrigeration, *en route* from the factory to remote clinics. It cools via a water–filled chamber with a block of ice on top, providing stable and energy–efficient temperatures that can be made with sporadic or solar power supplies. *Sure Chill* made more than 1000 fridges in 2014, supplying 45 countries – and customers include UNICEF. It is nearing WHO approval for its longest–lasting cooler. Peter Saunders, *Sure Chill*’s chairman, states that the Ebola epidemic highlights the urgency of improving global health infrastructure. The BMGF is a major investor in *Sure Chill*, giving more than US$ 1 million to help develop the coolers. (*Financial Times*, 9 Jan 2015)

➢ According to UNICEF, 14 million children are suffering hardship and trauma as a result of the war in Syria and Iraq, as it highlights the needs of children struggling to cope with violence and the global danger of failing to help a generation preyed on by extremist groups. A report from humanitarian agencies showed that the warring factions have ignored demands for access for humanitarian aid, and that the number of children needing aid has increased by one–third in a year, whilst funding for aid agencies has fallen sharply in relation to needs. Islamic State are increasingly recruiting children into active roles. Despite the chaos, UNICEF and other partners have vaccinated 2.9 million children against polio and 840 000 children against measles. However, the UN estimates that 4.8 million people, including 2 million children, are trapped in areas that cannot be reached by aid. It is dangerous to deliver aid across conflict lines, and security forces regularly remove surgical supplies from convoys. UNICEF has sought US$ 815 million to support its work in these countries, but has only raised 10% of that amount. (*New York Times*, 12 Mar 2015)

➢ UNICEF and private investors, including UBS and the Children’s Investment Fund Foundation have launched a US$ 1 billion independent fund (“The Power of Nutrition”) to tackle childhood and maternal malnutrition in some of the world’s poorest countries. It will focus on programmes that cover that first 1000 days of life, from maternal nutrition at conception to a child’s nutrition before s/he reaches school age. UNICEF estimates that 161 million children have stunted development due to poor nutrition – and under–nutrition contributes to 45% of deaths in children aged under 5 years. Properly nourished children are 33% more likely to escape poverty as adults. Sri Mulyani Indrawati of the World Bank called under–nutrition one of the world’s “most serious but least addressed” public health problems. “Getting children the right nutrients at the right time can save 3 million lives and make sure that children keep up in school and become productive adults.” (*USA Today*, 16 Apr 2015)

➢ In light of tragedies on the Mediterranean Sea, where hundreds of men, women and children are dead or missing, UNICEF calls for all actions to be guided in the best interests of children. Children who survive these crossings are often placed in unsafe and unsuitable conditions, and may be criminalised. This is in violation of the Convention of the Rights of the Child. UNICEF calls for children to be cared for in a safe place – not a detention facility – with access to education, health, social and legal services and full implementation of existing safeguards. The numbers attempting this crossing will increase as summer approaches, and more senseless deaths could happen unless decisive action is taken. This includes following the EU’s existing safeguards for unaccompanied minors, and tackling the root causes of migration in the countries of origin – violence, poverty and discrimination – to avoid more tragedies. (*UNICEF*, 20 Apr 2015)

➢ Ahead of the 2015 World Immunisation Week, UNICEF praised China’s achievement in vaccinating millions of children, and called for all children to be vaccinated. An estimated 20% of children miss basic, life–saving, vaccinations, and the World Immunisation Week appeals for the poorest and most marginalised children to be reached. China’s Expanded Programme on Immunisation has contributed to steep falls in deaths and disability from preventable diseases (eg, polio, diphtheria and meningitis), and its reaching the MDG of reducing mortality in children under 5 years. Failure to vaccinate groups of children can cause outbreaks of preventable diseases that threaten all children. Therefore, this year’s focus includes dispelling some of the myths surrounding vaccination. UNICEF calls for all governments to provide adequate funding for vaccination, and to make it an essential part of universal health coverage. China has committed US$ 5 million to GAVI to support vaccination in the poorest parts of the world. (*UNICEF/Women of China*, 27 Apr 2015)

## WHO

➢ Dr Margaret Chan, the WHO director–general, announced that she would like all tobacco companies to be shut down. Speaking at the World Conference on Tobacco and Health in Abu Dhabi, she said that cigarette companies use various tactics to undermine anti–tobacco laws, including political funding. Governments are also being intimidated by industry’s litigation threats – although Bill Gates and Michael Bloomberg have announced an anti–tobacco litigation fund to help counteract these threats. However, the WHO notes that globally tobacco use is falling, and non–smoking is becoming the norm. It recommends that governments increase their efforts against industry efforts to buck these trends. (*Voice of America*, 18 March 2015)

➢ The WHO launched a network of rapid response medical teams to respond to humanitarian emergencies world–wide. It is appealing to health services and medical aid agencies to register with it, to enable the optimal co–ordination of specialist expertise and knowledge shearing. Dr Ian Norton, who heads the project, said project aims to have suitably qualified teams across the world ready to deploy in the case of major emergencies such as epidemics, earthquakes and tsunamis. “We are asking organisations, countries and NGOs to register. We expect at least 150 to register within the first year,” he said. Authorities in countries hit by emergencies will be able to consult the list and decide which foreign medical teams they need. (*AFP*, 8 Apr 2015)

➢ In a statement, the WHO admitted to “serious failings” in its handling of the Ebola crisis, and promised reforms to avoid a repeat. The statement listed 8 lessons learned from the outbreak, including improved communications and information–sharing. It is claimed that the WHO’s hesitancy in declaring the outbreak an emergency is a major factor behind the epidemic becoming the worst Ebola crisis on record, with more than 25 000 cases and 10 000 deaths. “We have learned lessons of humility. We have seen that old diseases in new contexts consistently spring new surprises,” said the WHO Director–General Margaret Chan. (*Reuters*, 20 April 2015)

➢ The WHO has called for the disclosure of all clinical trial results for new drugs, regardless of their success, to increase transparency in the drug innovation process. Many studies show that clinical trial results are never published, especially if they fail to show a beneficial impact – causing research duplication and money being wasted. Lack of access to existing data can hinder the development of drugs and vaccines for developing countries, as trials have to start from scratch and are therefore more expensive – and affects decision–making on international health initiatives. The medical charity Médecins Sans Frontičres (MSF) calls for increased disclosure of results, enforced by law and regulation if necessary. MSF notes that commercial confidentiality is insufficient reason for non–disclosure. “Once a drug or vaccine is registered and on the market, all data can be published,” says MSF’s Manica Balasegaram. (*scidev.net*, 23 Apr 2015)

➢ An independent panel on the WHO response to Ebola states that the crisis could have been prevented if the WHO more quickly sought outside help. The panel was commissioned by WHO and will present its final report in July. The panel is also investigating why early warnings did not lead to an adequate response with international mobilisation and consistent communication strategies, and the delay in declaring Ebola a public–health emergency. The panel highlights the “serious gaps” in handling the outbreak, when traditional burial customs helped spread Ebola, and “bleak” communications reduced communities’ willingness to engage. The panel calls for extra investment in the WHO to strengthen its emergency–response capabilities as there is “strong, if not complete, consensus that WHO does not have a robust emergency operations capacity or culture.” (*Wall Street Journal*, 11 May 2015)

